# Identification of Molecular Subtypes in Head and Neck Squamous Cell Carcinoma Based on Dysregulated Immune LncRNAs

**DOI:** 10.1155/2022/9702789

**Published:** 2022-01-25

**Authors:** Yucong Du, Zhenhua Ji, Jianchun Liao, Huanhai Liu, Hu Peng

**Affiliations:** Department of Otorhinolaryngology-Head & Neck Surgery, Changzheng Hospital, Second Military Medical University, Shanghai 200003, China

## Abstract

Long noncoding RNAs (lncRNAs) perform indispensable functions in cancer pathologies and are involved in the onset and progression of multiple cancers. Multiple platforms were performed to comprehensively analyze the head and neck squamous cell carcinoma (HNSCC) for determining molecular subtypes. Molecular subtypes were clustered and analyzed by the “ConsensusClusterPlus” R package. The Limma software was utilized to screen for differentially expressed genes (DEGs). Functional enrichment analyses, including Gene Set Enrichment Analysis (GSEA), Kyoto Encyclopedia of Genes and Genomes (KEGG), and Gene Ontology (GO), were performed on the three database resources. Seventeen lncRNAs were determined as HNSCC-specific immune lncRNAs that were dysregulated. Our research identified and redefined two distinct molecular subtypes, C1 (230 samples) and C2 (269 samples). Moreover, the C1 subtype had a higher survival rate than the C2 subtype in HNSCC samples, as well as a prolonged median survival duration with activated immune response. 1531 DEGs, including 529 upmodulated genes and 1002 downmodulated genes, were identified in the above two subtypes. Functional enrichment analysis revealed that upmodulated genes in C2 were associated with tumorigenesis and development, while downregulated genes in C2 were associated with immune response. By comparing with the existing immunophenotyping group, it found that C1 had more overlaps with the existing Atypical and Basal, and C2 and Classical and Mesenchymal had a high degree of coincidence. On the basis of lncRNA, there were significant differences in the aspect of prognostic and immunological characteristics in the two identified molecular subtypes of HNSCC.

## 1. Introduction

Head and neck tumors have ranked sixth among the most common kind of malignant tumors worldwide, accounting for 16–40% of all malignancies [1]. About 90 percent of head and neck tumors are pathological like head and neck squamous cell carcinoma (HNSCC). At present, the incidence and death rates of HNSCC are increasing; there are 500,000 new cases worldwide every year, posing a serious threat to people's health. Epidemiological studies have shown that the main causes of HNSCC are tobacco and alcohol, and it tends to occur in males older than 40 years old [2]. In recent years, it has been shown that human papillomavirus infection is a significant risk factor in individuals with no history of alcohol consumption or smoking [3]. Despite the continuous improvement and maturation of treatment methods, the prognosis of HNSCC patients is still far from satisfactory, with only about a 40%∼50% 5-year survival rate due to the high risk of recurrence and metastasis [4].

Long noncoding RNAs (lncRNAs) are well-recognized noncoding RNAs that exceed 200 nucleotides in length [5]. So far, the study of lncRNA has just started in the primary stage. Along with the research on lncRNA unceasingly thorough, the regulatory role of lncRNA in dose-compensation, epigenetic, cell cycle, and cell differentiation was gradually discovered, and thus it further attracted more people's attention and made it to be an emerging hotspot in genetics. Some evidence demonstrated that lncRNAs played indispensable roles in the regulation of innate immune response, as well as more complex adaptive immune response and immune cell development [6, 7]. In addition, lncRNAs are associated with tumor microenvironment (TME) regulation and also perform a critical function in the formation of a heterogeneous and complex environment, which were filled with infiltrating immune cells and stromal cells [8–10]. High-expressed IR155HG is associated with high infiltration of immune cell subsets in ovarian cancer [11]. LncRNA HOTTIP upregulates the PD-L1 expression in Neutrophils, promotes immune evasion, and suppresses tumor immunotherapy and T cell proliferation [12]. It is a complex process about the participation of lncRNA in the aspect of immune regulation, and many crux immunomodulatory lncRNAs have not been discovered yet. Hence, it is crucial to find and detect novel immune-related lncRNAs and examine their function on HNSCC.

In our research, a coexpression network on the basis of the immune-related lncRNAs and mRNAs was firstly formed, and we obtained 17 lncRNAs associated with the immune and prognosis. Next, we identified two immune-related molecular subtypes of lncRNA in HNSCC samples. Moreover, analysis of their functional enrichment and characteristics of the immune microenvironment was performed, and the relationship between molecular subtypes and known molecular subtypes was investigated.

## 2. Materials and Methods

### 2.1. Data Collection and Processing

**Figure 1:**
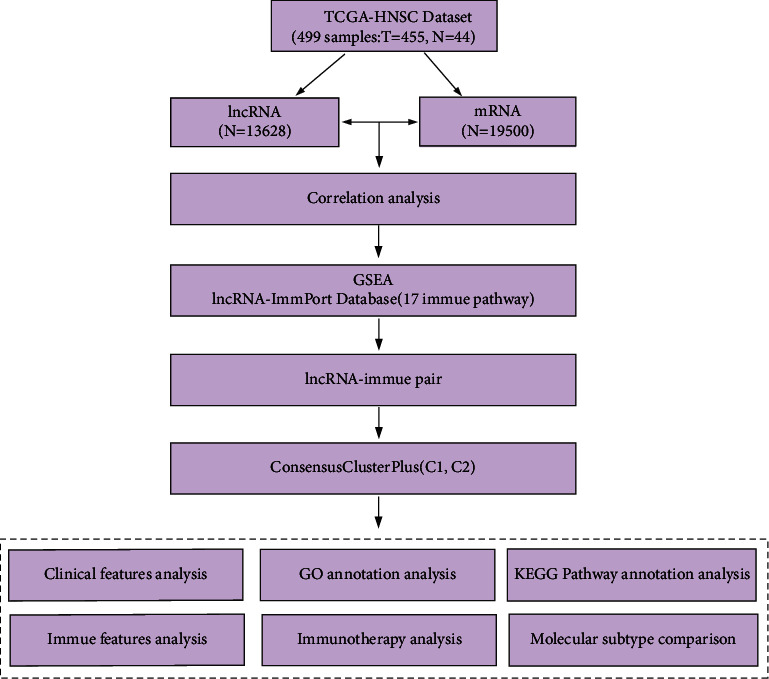
Work flowchart.

On April 20, 2020, we obtained HNSCC patients' data from the TCGA GDC API (https://portal.gdc.cancer.gov/) database containing their latest lncRNA expression level and clinical follow-up information [13]. In the TCGA dataset, we firstly used gene annotation of the GTF (V32 version) file in GENECODE (https://www.gencodegenes.org/human/) to divide the expression spectrum into two parts of lncRNA and mRNA and then converted the Ensembl IDs of these genes into Symbol form. In the TCGA-HNSCC dataset, we firstly deleted those genes with 0 expression values in HNSCC samples to maintain those gene sets with biological significance. Then, we obtained 13,628 lncRNAs' expression profiles and 19,500 protein-coding genes altogether. The following steps were used to process the TCGA RNA-seq data. Those samples without expression spectrum, clinical follow-up information, survival data, and survival status were all completely eliminated. We only stayed the solid tumor samples. The overall work flowchart of the study is shown in Figure 1.

### 2.2. Immune Function-Related Pathways

The ImmPort database [14] is comprised of a great amount of immune-related genes; it is extensively applied in immune-related studying. There are altogether 17 kinds of immune function-related pathways, which included Interleukins, Antimicrobials, Interferon Receptors, Cytokines, and Cytokine Receptors, TGFb Family Members, BCR Signaling, Interferons, Antigen Processing and Presentation, Interleukin receptor, Chemokines, Chemokine Receptors, TGFb Family Members, TCR Signaling Pathway, Natural Killer Cell Cytotoxicity, and TNF Family Members. Above all, signaling pathways consist of 1,811 kinds of related protein-coding genes.

### 2.3. Coexpression Analysis

Pearson correlation calculation approach was conducted to evaluate the association between the levels of mRNA and lncRNA expression in the HNSCC samples and to further study the coexpression association and function of the above two genes. For accuracy, those genes with a TPM expression value of 0 contained in the HNSCC tissues have been deleted completely. Moreover, to ensure that our calculation of the expression value complies with the rules of the normal distribution, we treated the expression spectrum in the HNSCC samples with log2 conversion. Then we used R language to calculate the Pearson correlation coefficients and significance *p* values in the HNSCC tissues-related mRNA and lncRNA. Eventually, all analyses showed that per lncRNA and 788 mRNAs altogether made obvious relevance in normal control samples, while per lncRNA and 92 mRNAs altogether made obvious relevance in the head and neck cancer tumor samples, based on the cutoff of |*R*| > 0.5 and *p* < 0.05.

### 2.4. Identification of Immune Function LncRNA Modulators

In order to figure out whether lncRNA connected with immune function, we detected the enrichment connection between lncRNA and 17 kinds of pathways that were involved in immune function by the GSEA method [15]. We acquired the score value, which can effectively stand for the correlation of mRNA and lncRNA by combining the expression correlation coefficient *p* value and *R*-value of lncRNA and mRNA in HNSCC and the corresponding normal control samples. The calculation equation is as follows: Score = −log10*P* × sign (*R*).

Based on the connection of lncRNA and mRNA, according to the correlation scores calculated by the above equation, all related mRNAs were arranged in ascending order from small to large for every lncRNA. At the same time, based on the connection of lncRNA and immune function, the GSEA method was performed to exactly compute the enrichment significance between every lncRNA and every immune function-related pathway, as well as the lncRES scores between the above two items [16]. On the premise of meeting the requirement of FDR < 0.05 and |lncRES| > 0.995, in accordance with all the above analysis, we got lncRNA, which effectively regulated immune function in HNSCC samples.

### 2.5. Enrichment Assessment of Dysregulated Immune-Related LncRNA and Cells

For analyzing the degree of enrichment between dysfunctional lncRNAs related to immune function in HNSCC samples and different immune cells, we gathered 24 kinds of immune cells marker gene collection by the ImmuCellAI, including Gamma_delta, CD8_naive, Neutrophil, Cytotoxic, Macrophage, Tr1, B cell, iTreg, MAIT, Th2, Effector_memory, Tfh, Central_memory, Th1, NKT, nTreg, DC, Exhausted, Monocyte, CD4_naive, NK, Th17, CD8_T, and CD4_T. Then, we extracted those aberrant immune lncRNAs, which obviously correlated to marker genes of 24 different immune cells, and further analyzed the obvious enrichment connection between extracted aberrant immune cells and immune lncRNAs by the method of the hypergeometric enrichment analysis. The calculation formula for the hypergeometric is as follows:

(1) $$\begin{array}{c} Pij=\frac{C_{M}^{k}C_{N-M}^{n-k}}{C_{N}^{n}}. \end{array}$$

The meanings of every alphabet were described detailed as follows: *i* denotes the enrichment value of lncRNA marker genes; *j* denotes immune cell; *n* denotes the number of mRNAs obviously connected with lncRNA in HNSCC samples; *M* denotes the number of marker genes present in immune cells; *K* denotes the quantity of obviously connection genes between immune cell and lncRNA; and *N* denotes 19,498, total mRNAs. Moreover, it will be regarded as having no significance when the *K* value is less than 3. On the basis of the calculation formula, the expression connection of each potential lncRNA and 24 immune cells and enrichment significance were calculated, and all candidates' aberrant immune lncRNA regulatory factors and immune cells' dramatically enrichment connection were also identified on the basis of the threshold *p* < 0.05. Eventually, we filtered out aberrant immune lncRNAs specific to HNSCC, which were apparently enriched in at least 12 immune cells.

### 2.6. Detection of Molecular Subtypes

In accordance with the aberrant immune lncRNAs' expression values in cancer samples, HNSCC samples were classified by the ConsensusClusterPlus [17]. We did the unsupervised clustering to the samples as well as determined the quantity of clustering by using the R-packets.

### 2.7. Survival Analysis of Molecular Subclasses

In accordance with the survival time (disease-specific survival: DSS, progression-free survival: PFS, and overall survival: OS), as well as the survival status in different sample categories, the samples were subjected to survival analysis. The survival rate, as well as the median survival time, was estimated by the method of Kaplan-Meier [18]. At the same time, on the basis of the log-rank test, the differences between distinct sample subtypes were gained through comparison among the groups [19].

### 2.8. Functional Enrichment and Differential Genes Analysis of Molecular Subtypes

The Limma software was employed to detect the DEGs in different molecular subtypes [20]. The screening was performed using FDR < 0.05 and |FC| > 1.2 as thresholds, and genes that met these requirements were selected and considered as DEGs for further analysis. On the basis of FDR < 0.05 as the threshold of significant enrichment, functional enrichment analysis of GO and KEGG utilizing the R software package WebGestaltR (http://www.webgestalt.org/option.php) [21] for differential genes was performed. GSEA was used for functional aggregation analysis of the expression profiles of molecular subtypes. |ES| > 0.4, *p* < 0.05, and FDR < 0.25 were used as the threshold for screening.

### 2.9. Analysis of Properties of Molecular Subclasses

For figuring out the differences of biological characteristics in various cancer samples, various characterizations of TCGA-HNSCC samples were acquired from existing articles [22], which included essential immunological molecular characteristics such as Atypical, Basal, Classical, and Mesenchymal. Meanwhile, Microenvironment Cell Populations (MCP) counter [23], Tumor Immune Estimation Resource (TIMER) [24], and Estimation of Stromal and Immune cells in MAlignant Tumor tissues using Expression data (ESTIMATE) [25] were all used to evaluate the immune scores in HNSCC samples. Additionally, the Wilcox rank-sum test was utilized to compare biological traits and characterize subtypes of diverse samples.

## 3. Results

### 3.1. Screening of Dysregulated Immune LncRNAs Specific to HNSCC

**Figure 2:**
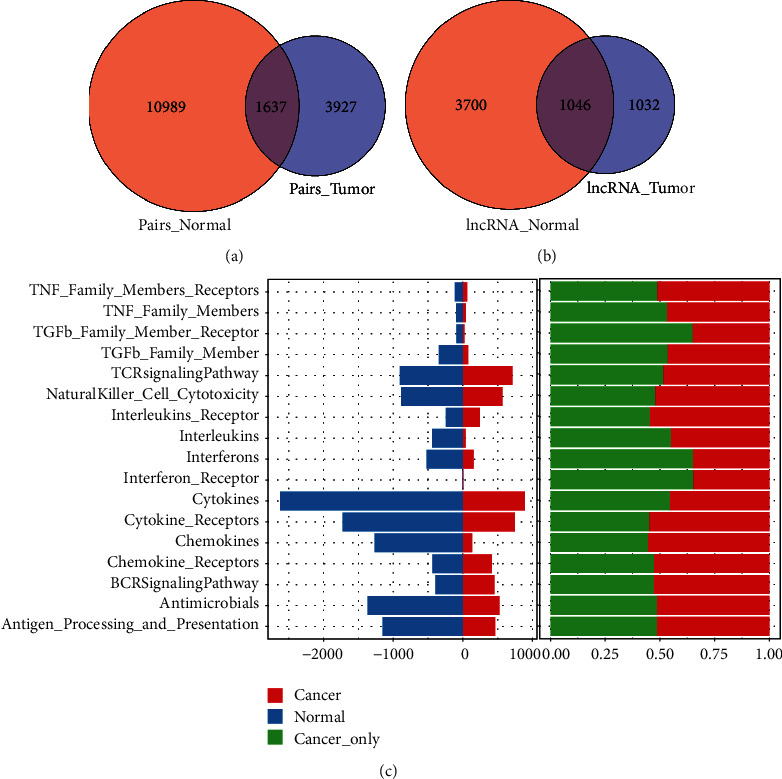
Identification of HNSCC-specific dysregulated immune lncRNAs. (a) LncRNA-immune pathway pairs were detected in tumor and normal samples. (b) LncRNAs were detected in both tumor and normal samples. (c) LncRNAs were enriched by immune function pathway in normal samples and tumor samples (LncRNAs enriched in normal and tumor samples in different pathways are shown on the left, and the proportion of tumor-specific enriched lncRNAs in the immune pathway of all tumor samples in the immune pathway is shown on the right).

**Figure 3:**
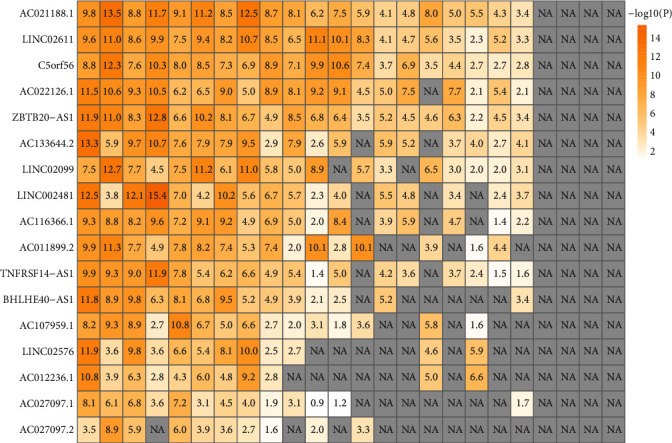
Enrichment significance of dysregulated immune cells and immune lncRNA.

Under the conditions of FDR < 0.05 and |lncRES| > 0.995, we identified 12626 important relationship pairs between immune-related lncRNA and immune function pathway in normal samples, including 4746 lncRNA regulatory factors, which were enriched in different immune function sets. Moreover, we also identified 5564 important relationship pairs between immune-related lncRNA and immune function pathways in HNSCC samples, including 2078 lncRNA regulatory factors, which were enriched in different immune function sets. In normal samples and tumor samples, only 1637 pairs of lncRNA-immune function pathway relationship were shared, which accounted for only 12.97% in normal samples (1637/12626) and only 29.42% in tumor samples (1637/5564), which showed that the lncRNA-immune function pathway relationship between normal samples and tumor samples was very different (Figure 2(a)). Similarly, there were 1046 immune-related lncRNAs in the intersection of normal samples and tumor samples, accounting for 22.04% (1046/4746) in normal samples and 50.34% (1046/2078) in tumor samples (Figure 2(b)). Among the tumor samples, 1032 (49.66%, 1032/2078) lncRNAs were immune-specific lncRNAs, serving as the gene set of tumor-specific immune-related lncRNAs. In addition, a higher proportion of HNSCC-specific immune function-related lncRNA performed a function in the modulation of immune-related molecular pathways such as TGFb_Family_Member_Receptor, Interferons, and Interferon_Receptor (Figure 2(c)). The connection between lncRNA and immune cells was analyzed by a hypergeometric test. On the premise of meeting the requirement of *p* < 0.05, 2616 important lncRNA-immune cell pairs were determined, and 17 lncRNAs considerably enriched in a minimum of 10 immune cells were further screened out as lncRNA collections for the immune dysregulation in HNSCC (Figure 3).

### 3.2. Identification of Molecular Subtypes of HNSCC

**Figure 4:**
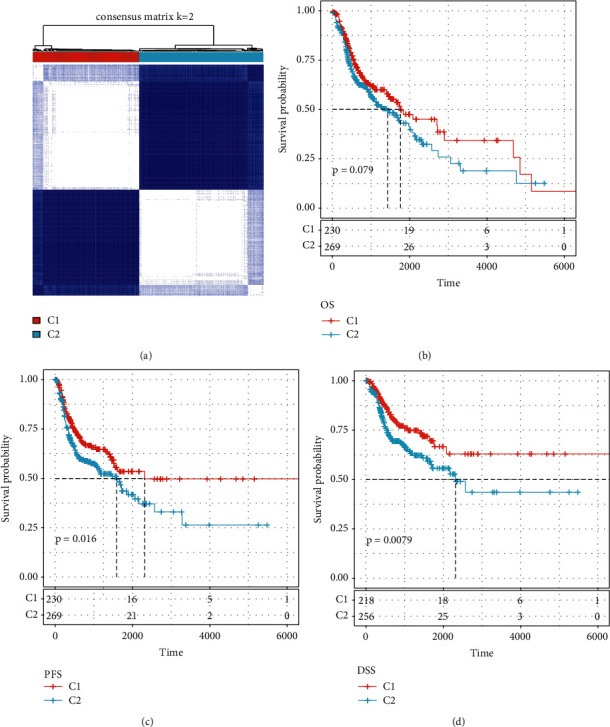
Detection of molecular subtypes of HNSCC. (a) Two molecular subtypes were identified in HNSCC. (b) Overall survival times in two molecular subtypes. (c) PFS times in two molecular subtypes. (d) DSS times in two molecular subtypes.

It has been reported that the subclassification of tumor tissues is of valuable reference for individualized therapy of head and neck cancer. Considering the above reason, we classified the HNSCC tissues by detecting the expression of 17 HNSCC-specific immune lncRNAs. According to the R package ConsensusClusterPlus, the head and neck cancer samples obtained from the TCGA database were assigned into two categories, namely, C1 containing 230 samples and C2 containing 269 samples (Figure 4(a); Supplementary Table S1). Additionally, the result of HNSCC samples from TCGA by survival analysis showed borderline significant (*p*=0.079) on OS (Figure 4(b)) and significant survival differences on PFS as well as DSS (*p*=0.016; *p*=0.0079) (Figures 4(c) and 4(d)), with HNSCC samples in the C1 subtype having a longer median survival time and a higher survival rate.

### 3.3. Differential Analysis of Molecular Subtypes in Immune Cell Score

**Figure 5:**
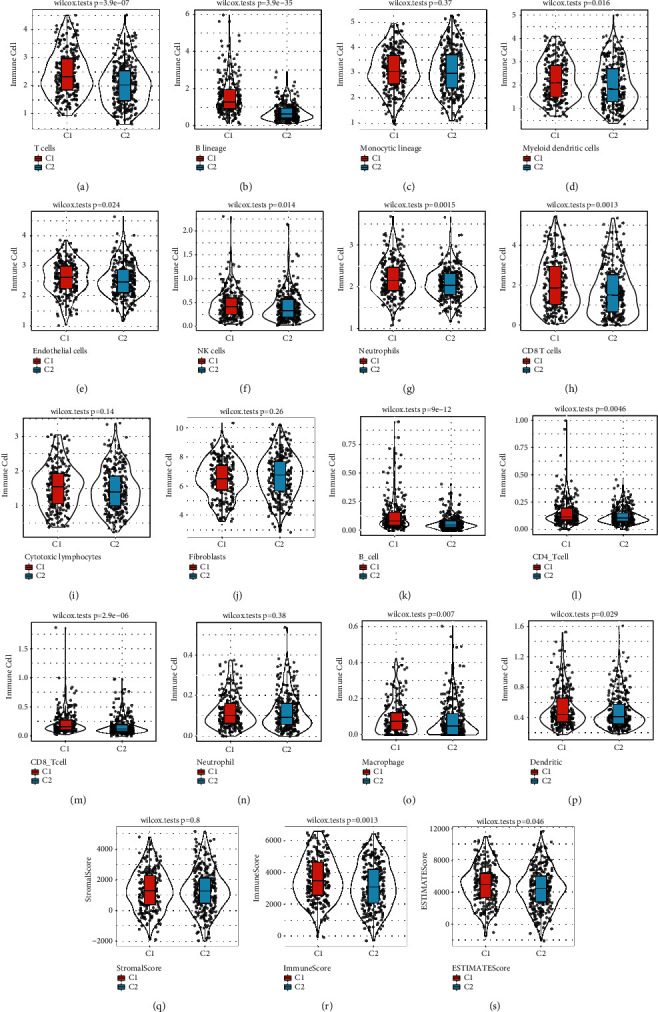
Differential analysis of molecular subtypes in immune cell score. (a) The scores of T cells between the 2 molecular subtypes. (b) The scores of B lineage between the 2 molecular subtypes. (c) The scores of monocytic lineage between the 2 molecular subtypes. (d) The scores of Myeloid dendritic cells between the 2 molecular subtypes. (e) The scores of endothelial cells between the 2 molecular subtypes. (f) The scores of NK cells between the 2 molecular subtypes. (g) The scores of Neutrophils between the 2 molecular subtypes. (h) The scores of CD8 T cells between the 2 molecular subtypes. (i) The scores of cytotoxic lymphocytes between the 2 molecular subtypes. (j) The scores of fibroblasts between the 2 molecular subtypes. (k) The scores of B cells between the 2 molecular subtypes. (l) The scores of CD4 T cells between the 2 molecular subtypes. (m) The scores of CD8 T cells between the 2 molecular subtypes. (n) The scores of Neutrophils between the 2 molecular subtypes. (o) The scores of macrophages between the 2 molecular subtypes. (p) The scores of dendritic between the 2 molecular subtypes. (q) StromalScore scores between the 2 molecular subtypes. (r) ImmuneScore scores between the 2 molecular subtypes. (s) ESTIMATEScore scores between molecular subtypes.

Ten immune cells were utilized to compute the immune scores in the C1 and C2 molecular subtypes by MCPcounter. The results proved that the T cells, NK cells, CD8 T cells, B lineage, Myeloid dendritic cells, and Neutrophils were different in the 2 molecular subtypes mentioned above (*p* < 0.05) (Figures 5(a)–5(j)), and the scores of immune cells were greater in C1 molecular subtype as opposed to those in C2 molecular subtype. TIMER was performed to determine the immune scores of the 6 kinds of immune cells in the C1 and C2 molecular subtypes, of which CD4 T cells, B cells, CD8 T cells, dendritic, and macrophage differed in the above 2 molecular subtypes (*p* < 0.05) (Figures 5(k)–5(p)), and the scores of immune cells were also greater in C1 molecular subtype as opposed to those in C2 molecular subtype. Next, ESTIMATE was used to calculate StromalScore, ImmuneScore, and ESTIMATEScore, and ImmuneScore and ESTIMATEScore were obviously distinct in the 2 molecular subtypes (*p* < 0.05) (Figures 5(q)–5(s)), and ImmuneScore was higher in C1 subtypes than that of C2 subtype sample ImmuneScore. These data indicated that the C1 subtype had stronger immunity, which might account for a better prognosis of the C1 subtype.

### 3.4. Screening of DEGs and Functional Enrichment Analysis

**Figure 6:**
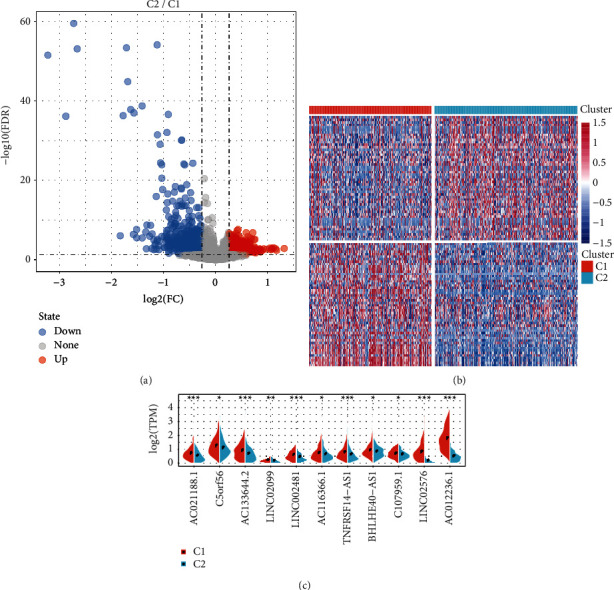
Detection of DEGs. (a) Volcanogram of differential genes among molecular subtypes. (b) Heatmap of differential genes between TCGA molecular subtypes. (c) The expression differences of 11 immune-related lncRNAs in molecular subtypes. ^*∗*^*p* < 0.05, ^*∗∗*^*p* < 0.01, and ^*∗∗∗*^*p* < 0.001.

**Figure 7:**
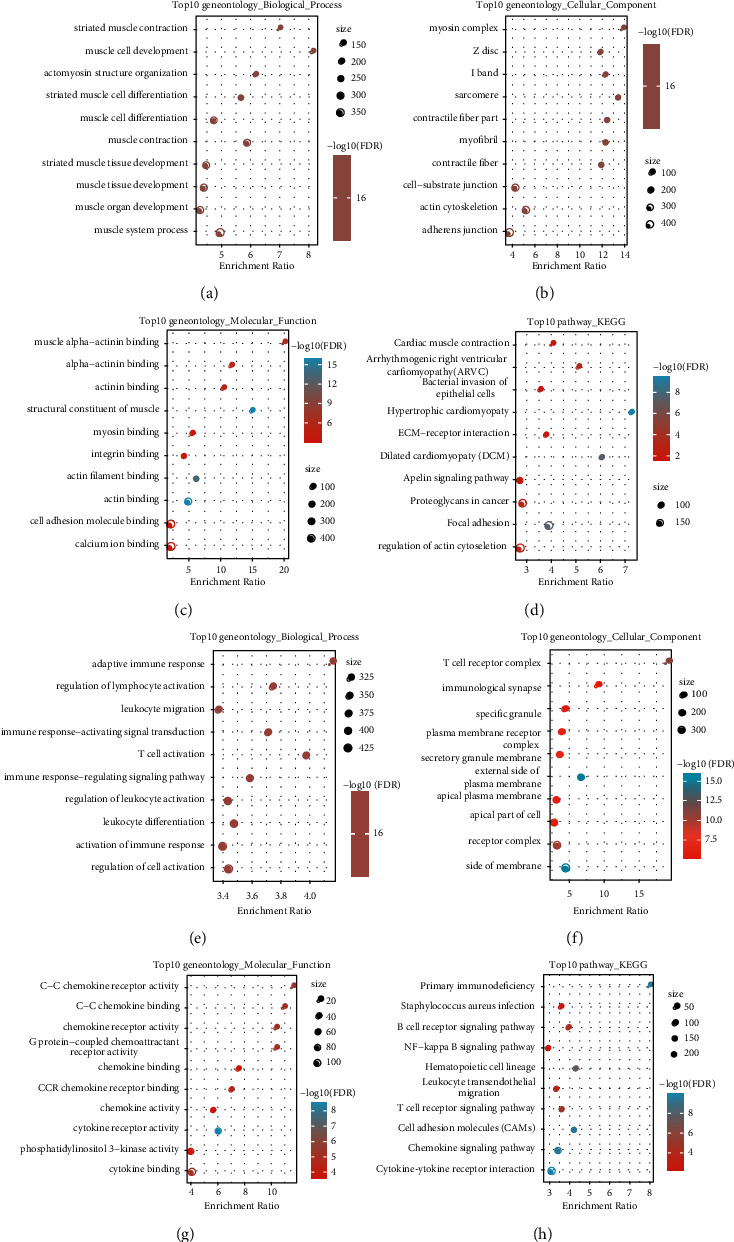
Detection and functional enrichment analysis of DEGs. (a) Top10 outcomes of biological process enrichment of differentially upmodulated genes in C2 molecular subtypes. (b) Top10 outcomes of cellular component enrichment of differentially upregulated genes in C2 molecular subtypes. (c) Top10 outcomes of molecular function enrichment of differentially upregulated genes in C2 molecular subtypes. (d) KEGG enrichment of differentially upregulated genes in C2 molecular subtypes. (e) Top10 outcomes of biological process enrichment of differentially downregulated genes in C2 molecular subtypes. (f) Top10 outcomes of cellular component enrichment of differentially downregulated genes in C2 molecular subtypes. (g) Top10 results of molecular function enrichment of differentially downregulated genes in C2 molecular subtypes. (h) KEGG enrichment of differentially downregulated genes in C2 molecular subtypes.

DEGs between C1 and C2 molecular subtypes were identified by the Limma (3.40.6) package. There were altogether 1531 DEGs; specifically, 1002 genes were downmodulated, and 529 genes were upmodulated (Figures 6(a) and 6(b)). The differential expression of 17 immune-related aberrant lncRNAs was compared in the 2 different molecular subtypes. The results indicated that, in the two molecular subtypes, 64.71% lncRNA (11/17) had significant differential expression (*p* < 0.05), and there were apparently higher expression levels of these lncRNAs in the C1 subtype than those in the C2 subtype (Figure 6(c)). The R software package WebGestaltR was conducted to do the analysis of GO and KEGG functional enrichment on the differentially upregulated 529 genes (0.4.3). In the 529 genes, under the conditions of FDR, <0.05, 240 pathways were annotated to biological process (BP) (Figure 7(a)), 68 pathways were annotated to cellular component (CC) (Figure 7(b)), and 36 pathways were annotated to molecular function (MF) (Figure 7(c)). A total of 12 KEGG pathways with significant differences and Proteoglycans in cancer, ECM-receptor interaction, and Focal expression tumor development pathways were obviously enriched (Figure 7(d)). The R software package WebGestaltR was also conducted to do the analysis of KEGG and GO functional enrichment on the differentially downregulated 1002 genes. In the 1002 genes, under the conditions of FDR <0.05, there were 93 pathways annotated to BP (Figure 7(e)), 59 pathways annotated to CC (Figure 7(f)), 40 pathways annotated to MF (Figure 7(g)), and a total of 15 KEGG pathways with significant differences (Figure 7(h)), including the immune-related pathways, such as the primary immunodeficiency, the signaling pathway of B cell receptor, NF-kappa B, and T cell.

### 3.5. GSEA Analysis

**Figure 8:**
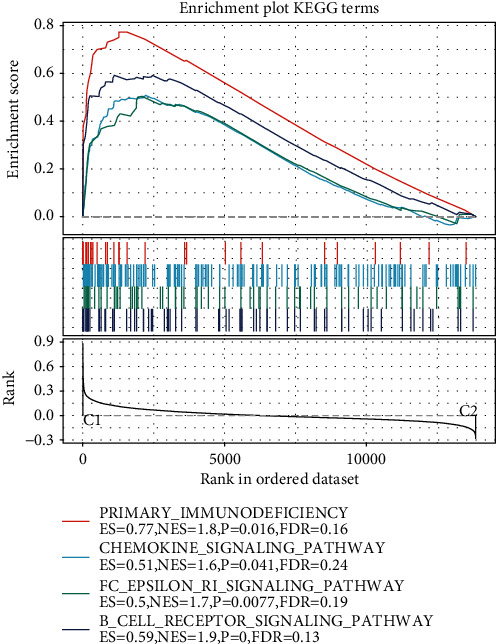
GSEA analysis. GSEA analysis of molecular subtypes in TCGA dataset.

GSEA was used to analyze pathways that were significantly enriched by molecular subtype C1 and subtype C2, and it was found that immune-related B_CELL_RECEPTOR_SIGNALING_PATHWAY, CHEMOKINE_SIGNALING_PATHWAY, FC_EPSILON_RI_SIGNALING_PATHWAY, and PRIMARY_IMMUNODEFICIENCY were significantly enriched by the C1 subtype (*p* < 0.05, FDR <0.25) (Figure 8). The results also proved that molecular subtype C1 had stronger immunity.

### 3.6. Gene Expression Analysis and Immunotherapy Gene Difference Analysis

**Figure 9:**
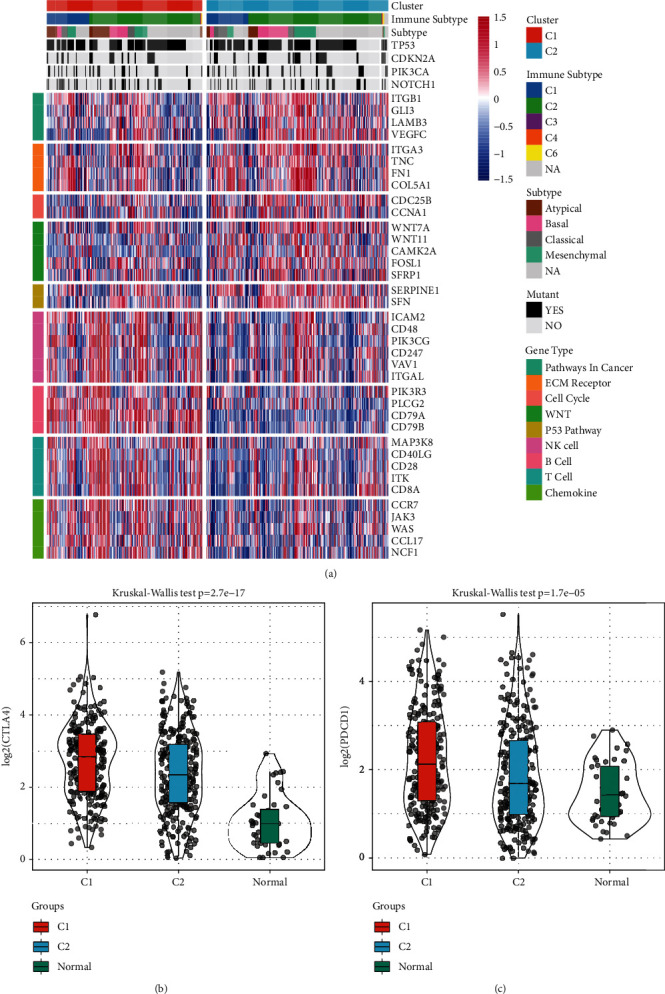
Gene expression analysis and immunotherapy gene difference analysis. (a) Heatmaps of marker genes of molecular subtypes in the TCGA dataset. (b) The expression of CTLA4 in the C1 molecular subtype was elevated as opposed to that in the C2 molecular subtype. (c) PDCD1 expression in the C1 molecular subtype was higher than that in the C2 molecular subtype. ^*∗*^*p* < 0.05, ^*∗∗*^*p* < 0.01, and ^*∗∗∗*^*p* < 0.001.

Pathway genes related to tumor development and immunity were selected from the gene set for c2.cp.kegg.v7.0.symbols. Then, we compared the expression level of those selected genes in the 2 molecular subtypes; the results indicated that the level of tumor-related pathway genes expression for EMC, Cell Cycle, WNT, and P53 was substantially elevated in the C2 subtype in contrast with the C1 subtype expression, while immune-related NK Cell, B Cell, T Cell, and Chemokine pathway genes were considerably elevated in the C1 subtype in contrast with the C2 subtype (Figure 9(a)). Immunotherapy, as a tumor therapy, had been validated in a variety of tumors, among which CTLA4 and PDCD1 were the most studied. We compared the expression of CTLA4 and PDCD1 among molecular subtype and normal sample and found that the expressions of CTLA4 and PDCD1 in the normal sample were lower than those in the tumor sample, while in the C1 subtype, the expressions of these two genes were higher than those in the C2 subtype in the tumor sample (Figures 9(b) and 9(c)).

### 3.7. Comparison with Existing Molecular Subtypes

**Figure 10:**
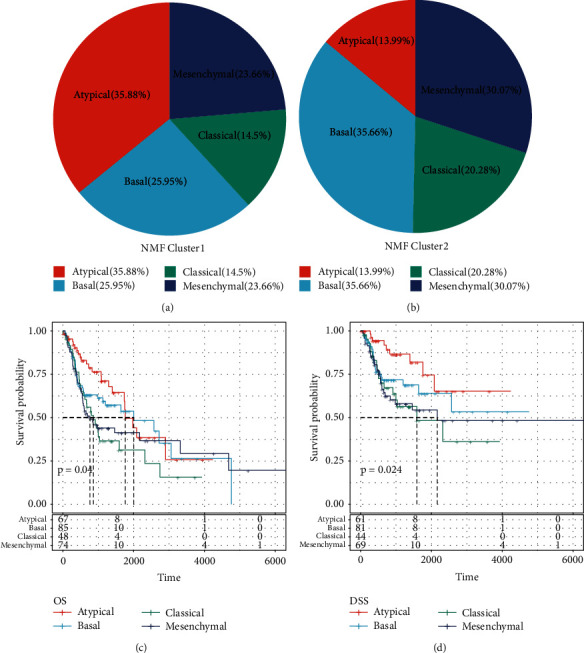
Comparison with known molecular subtypes. (a) Distribution of C1 molecular subtypes in four immune subtypes (Atypical, Basal, Classical, and Mesenchymal). (b) Distribution of C2 molecular subtypes in four immune subtypes (Atypical, Basal, Classical, and Mesenchymal). (c) Overall survival KM curves of four immune subtypes (Atypical, Basal, Classical, and Mesenchymal). (d) Disease-specific survival KM curves of four immune subtypes (Atypical, Basal, Classical, and Mesenchymal).

Compared with the existing molecular subtypes Atypical, Basal, Classical, and Mesenchymal, what was interesting was that we found that the proportion of Atypical and Basal in the C1 subtype samples has increased significantly, accounting for 61.38%, which was related to a good prognosis; the C2 subtype contains Atypical and Basal, accounting for only 49.56%, which were also the reason for the poor prognosis of C2 (Figures 10(a) and 10(b)). These four molecular subtypes had dramatic differences in OS and DSS. Among them, the Atypical and Basal subtypes had a better prognosis, while the Classical and Mesenchymal subtypes had poor prognoses (Figures 10(c) and 10(d)).

## 4. Discussion

TCGA is an outstanding database, and it attempted to use genome analysis technology to map out the genome variation maps of all human cancers and conduct systematic analysis. At present, TCGA has performed various crucial molecular characterizations of unique histological cancer types, including HNSCC [26, 27]. A unique PRCC subtype with the characterization of the CpG island methylation phenotype (CIMP-RCC) and poor survival rate was identified, as well as the early onset of the fumarate hydratase (FH) gene [28]. A previous study applying cluster analysis on multiplatform genomic and genetic data to compare all available kidney tumor samples with histological types in TCGA showed that most histological subtypes could be reconstructed [29]. In our research, the TCGA database was determined to study the molecular typing of lncRNAs for HNSCC and identified two molecular subtypes with significant prognostic differences.

Increasing amounts of evidence show that lncRNA exerts an enormous function on tumor progression and tumorigenesis, including HNSCC, and immune-related lncRNA has been reported in tumor typing. Two different microenvironment-based subtypes with the characterization of exhausted or active immune response markers in HNSCC were identified [30]. ImmLnc can determine the priority of lncRNAs that were related to cancers, such as the identification of 3 molecular subtypes (proliferative, immunological, and intermediate) in non-small-cell lung cancer [16]. According to the lncRNA-TF-gene triplet, stratifying patients who suffer from cancers can help identification of distinct subtypes with different clinical characteristics, such as survival rates [31]. According to the expression of 143 kinds of lncRNAs, which were related to immune, renal clear cell carcinoma samples were divided into three immune clusters [32]. In our study, according to the analysis of R page ConsensusClusterPlus in lncRNA that was related to immune, we found and identified two prognostic clusters showing different survival potentials (C1 and C2). In the above two clusters, we comprehensively analyzed their immune cell types and immune characteristic scores. When compared with cluster 2, our data showed a higher score of response to TGF-beta and a lower score of response to IFN-gamma and wound healing in cluster 1. Moreover, in the above two molecular subtypes, there were significant differences in the score of Neutrophils, Myeloid dendritic cells, Endothelial cells, Monocytic lineage, Fibroblasts, and CD8 T cells. The analysis results elaborated that the enormous immunological differences between the 2 groups of molecular subtypes in HNSCC samples may be the main cause of dramatically different prognoses between the two groups.

Although there are important discoveries revealed by these results, there are still limitations. First, lncRNA needs to be researched with more methods and from more different aspects, which may offer more new therapeutic targets to treat patients who suffer from HNSCC. Secondly, it is not quite enough to just validate the relationship of immune-related lncRNA and HNSCC with independent patient data. Thus, it is absolutely critical to verify the effect of immune-related lncRNA in HNSCC with more patient datasets for accelerating clinical application.

## 5. Conclusions

Overall, we have identified two molecular subtypes that were closely related to clinical outcomes in HNSCC patients based on the immune lncRNA.

## Data Availability

The datasets utilized and/or evaluated during this study are accessible upon valid request from the corresponding author.

## Supplementary Materials

Supplementary Materials Supplementary Table S1: Molecular subtype information for each patient.
