# Supplementary material for: Identification of Molecular Subtypes in Head and Neck Squamous Cell Carcinoma Based on Dysregulated Immune LncRNAs
Source: J Oncol. 2022 Jan 25;2022:9702789. doi: 10.1155/2022/9702789 (PMC8808126; doi:10.1155/2022/9702789)
Supplement: Supplementary Materials — Supplementary Table S1: Molecular subtype information for each patient. [file 9702789.f1.pdf]

Supplementary Table S1：Molecular subtype infor

TCGA-CR-5250-01	C1
TCGA-CV-6942-01	C1
TCGA-CV-A463-01	C1
TCGA-CV-6436-01	C1
TCGA-CQ-A4CH-01	C1
TCGA-CQ-A4CE-01	C1
TCGA-WA-A7H4-01	C1
TCGA-F7-A620-01	C1
TCGA-DQ-5624-01	C1
TCGA-CQ-7071-01	C1
TCGA-CR-7371-01	C1
TCGA-CN-6992-01	C1
TCGA-D6-A74Q-01	C1
TCGA-CV-7263-01	C1
TCGA-BA-6870-01	C1
TCGA-MT-A7BN-01	C1
TCGA-BA-6873-01	C1
TCGA-CV-6959-01	C1
TCGA-CR-7377-01	C1
TCGA-CV-5430-01	C1
TCGA-CN-A497-01	C1
TCGA-CV-5978-01	C1
TCGA-CV-A468-01	C1
TCGA-CV-6952-01	C1
TCGA-D6-6826-01	C1
TCGA-TN-A7HI-01	C1
TCGA-UF-A7JJ-01	C1
TCGA-CR-6478-01	C1
TCGA-UF-A719-01	C1
TCGA-UF-A7JT-01	C1
TCGA-CR-7367-01	C1
TCGA-QK-A6IF-01	C1
TCGA-D6-A6EO-01	C1
TCGA-CN-A63T-01	C1
TCGA-D6-A4ZB-01	C1
TCGA-CR-7380-01	C1
TCGA-D6-6517-01	C1
TCGA-CV-7254-01	C1
TCGA-QK-A6IJ-01	C1
TCGA-CV-6935-01	C1
TCGA-CR-7379-01	C1
TCGA-CN-A498-01	C1
TCGA-BA-A6DL-01	C1
TCGA-CR-7370-01	C1
TCGA-CR-6484-01	C1
TCGA-CR-7390-01	C1
TCGA-CQ-5325-01	C1
TCGA-DQ-5629-01	C1
TCGA-BA-5559-01	C1
TCGA-CN-A63U-01	C1
TCGA-CV-7095-01	C1
TCGA-CN-5358-01	C1
TCGA-CN-A6UY-01	C1
TCGA-KU-A66S-01	C1
TCGA-CN-6017-01	C1
TCGA-UF-A71B-01	C1
TCGA-CV-7438-01	C1

TCGA-CV-7242-01	C1
TCGA-CN-A6V3-01	C1
TCGA-QK-A8ZA-01	C1
TCGA-QK-A6VC-01	C1
TCGA-BB-A5HZ-01	C1
TCGA-HD-7754-01	C1
TCGA-H7-A6C4-01	C1
TCGA-P3-A5Q5-01	C1
TCGA-CN-4740-01	C1
TCGA-CV-A45Q-01	C1
TCGA-CR-7392-01	C1
TCGA-D6-A6EK-01	C1
TCGA-CR-7374-01	C1
TCGA-CR-7369-01	C1
TCGA-CR-5248-01	C1
TCGA-CV-6939-01	C1
TCGA-CV-7415-01	C1
TCGA-HD-A4C1-01	C1
TCGA-BB-4223-01	C1
TCGA-CV-5443-01	C1
TCGA-CN-5374-01	C1
TCGA-CX-7086-01	C1
TCGA-D6-A4Z9-01	C1
TCGA-TN-A7HJ-01	C1
TCGA-BB-8601-01	C1
TCGA-CR-7368-01	C1
TCGA-CV-7101-01	C1
TCGA-BB-A5HU-01	C1
TCGA-MZ-A5BI-01	C1
TCGA-CV-A6JM-01	C1
TCGA-CN-6012-01	C1
TCGA-HD-8314-01	C1
TCGA-CR-6467-01	C1
TCGA-DQ-7591-01	C1
TCGA-HD-8635-01	C1
TCGA-QK-A8Z7-01	C1
TCGA-CV-5432-01	C1
TCGA-P3-A6T7-01	C1
TCGA-BA-6869-01	C1
TCGA-BA-5153-01	C1
TCGA-CV-A45U-01	C1
TCGA-CV-A45X-01	C1
TCGA-QK-AA3J-01	C1
TCGA-UF-A71E-01	C1
TCGA-QK-A8ZB-01	C1
TCGA-CV-6941-01	C1
TCGA-HD-7229-01	C1
TCGA-CV-6953-01	C1
TCGA-CN-4722-01	C1
TCGA-F7-A622-01	C1
TCGA-CV-7252-01	C1
TCGA-CN-4726-01	C1
TCGA-P3-A6T4-01	C1
TCGA-CV-6945-01	C1
TCGA-BA-A4IG-01	C1
TCGA-UF-A71D-01	C1
TCGA-IQ-A61I-01	C1
TCGA-CX-A4AQ-01	C1

TCGA-CN-6010-01	C1
TCGA-CN-4742-01	C1
TCGA-T2-A6WZ-01	C1
TCGA-CN-6018-01	C1
TCGA-CR-6487-01	C1
TCGA-CV-7248-01	C1
TCGA-CR-7395-01	C1
TCGA-IQ-A61E-01	C1
TCGA-CN-5360-01	C1
TCGA-CN-5356-01	C1
TCGA-CN-6021-01	C1
TCGA-CN-4723-01	C1
TCGA-CV-5441-01	C1
TCGA-CN-4733-01	C1
TCGA-CQ-6223-01	C1
TCGA-CN-4739-01	C1
TCGA-F7-A623-01	C1
TCGA-CV-A45T-01	C1
TCGA-CN-6996-01	C1
TCGA-CV-6943-01	C1
TCGA-CQ-6225-01	C1
TCGA-CR-5249-01	C1
TCGA-P3-A6SW-01	C1
TCGA-HD-7831-01	C1
TCGA-CV-7411-01	C1
TCGA-CV-7238-01	C1
TCGA-CR-7364-01	C1
TCGA-CN-6020-01	C1
TCGA-CV-5431-01	C1
TCGA-CV-6936-01	C1
TCGA-F7-7848-01	C1
TCGA-CR-7389-01	C1
TCGA-CV-5973-01	C1
TCGA-QK-A6II-01	C1
TCGA-CN-6023-01	C1
TCGA-BB-8596-01	C1
TCGA-KU-A6H7-01	C1
TCGA-CV-A45Y-01	C1
TCGA-D6-A6ES-01	C1
TCGA-CN-4729-01	C1
TCGA-IQ-A61H-01	C1
TCGA-CV-7418-01	C1
TCGA-CN-5355-01	C1
TCGA-CV-7424-01	C1
TCGA-MT-A67D-01	C1
TCGA-CN-4735-01	C1
TCGA-CV-5970-01	C1
TCGA-CV-A465-01	C1
TCGA-CV-A6JY-01	C1
TCGA-BA-A4II-01	C1
TCGA-IQ-7632-01	C1
TCGA-IQ-A6SH-01	C1
TCGA-BB-4217-01	C1
TCGA-CV-A460-01	C1
TCGA-UF-A7JF-01	C1
TCGA-CR-7385-01	C1
TCGA-CR-7394-01	C1
TCGA-CR-7388-01	C1

TCGA-CX-7085-01	C1
TCGA-CR-6470-01	C1
TCGA-CX-7219-01	C1
TCGA-UP-A6WW-01	C1
TCGA-CV-7180-01	C1
TCGA-H7-A76A-01	C1
TCGA-BA-A6DI-01	C1
TCGA-F7-A61V-01	C1
TCGA-QK-AA3K-01	C1
TCGA-QK-A8Z8-01	C1
TCGA-D6-6824-01	C1
TCGA-BA-5151-01	C1
TCGA-CV-7410-01	C1
TCGA-CR-6482-01	C1
TCGA-CN-4738-01	C1
TCGA-CN-6013-01	C1
TCGA-CR-6477-01	C1
TCGA-BB-A5HY-01	C1
TCGA-CV-A45P-01	C1
TCGA-DQ-5631-01	C1
TCGA-BA-A6DD-01	C1
TCGA-CR-6492-01	C1
TCGA-T3-A92M-01	C1
TCGA-4P-AA8J-01	C1
TCGA-CV-7440-01	C1
TCGA-BA-5152-01	C1
TCGA-CV-7261-01	C1
TCGA-HD-7753-01	C1
TCGA-CQ-5323-01	C1
TCGA-CR-6481-01	C1
TCGA-CV-7250-01	C1
TCGA-UF-A718-01	C1
TCGA-BA-5558-01	C1
TCGA-CV-6937-01	C1
TCGA-CR-5243-01	C1
TCGA-HD-A6HZ-01	C1
TCGA-CR-7404-01	C1
TCGA-CN-A6V6-01	C1
TCGA-CR-6474-01	C1
TCGA-D6-8568-01	C1
TCGA-D6-A6EQ-01	C1
TCGA-CQ-A4C9-01	C1
TCGA-CV-A45W-01	C1
TCGA-BA-A8YP-01	C1
TCGA-D6-6823-01	C1
TCGA-MZ-A6I9-01	C1
TCGA-BB-4224-01	C1
TCGA-F7-A50I-01	C1
TCGA-CR-6480-01	C1
TCGA-CN-5369-01	C1
TCGA-CR-7402-01	C1
TCGA-CN-5363-01	C1
TCGA-CQ-6228-01	C1
TCGA-UF-A7JD-01	C1
TCGA-T2-A6X0-01	C1
TCGA-CR-7373-01	C1
TCGA-CV-7435-01	C1
TCGA-CN-5361-01	C2

TCGA-CR-7391-01	C2
TCGA-CV-5436-01	C2
TCGA-CV-6441-01	C2
TCGA-CV-6948-01	C2
TCGA-CV-6962-01	C2
TCGA-F7-8489-01	C2
TCGA-CQ-5327-01	C2
TCGA-CV-7423-01	C2
TCGA-CV-7100-01	C2
TCGA-MZ-A7D7-01	C2
TCGA-CQ-A4CI-01	C2
TCGA-D6-6825-01	C2
TCGA-CV-7413-01	C2
TCGA-CX-7082-01	C2
TCGA-MT-A51X-01	C2
TCGA-CQ-5324-01	C2
TCGA-CV-6433-01	C2
TCGA-P3-A6T0-01	C2
TCGA-CR-7399-01	C2
TCGA-CQ-7065-01	C2
TCGA-CN-4741-01	C2
TCGA-CN-A499-01	C2
TCGA-CR-6473-01	C2
TCGA-CV-6003-01	C2
TCGA-IQ-A6SG-01	C2
TCGA-CN-4727-01	C2
TCGA-F7-A61S-01	C2
TCGA-CN-4725-01	C2
TCGA-C9-A480-01	C2
TCGA-CR-7393-01	C2
TCGA-BA-A6DG-01	C2
TCGA-CQ-6227-01	C2
TCGA-BB-A6UO-01	C2
TCGA-CQ-5329-01	C2
TCGA-P3-A6SX-01	C2
TCGA-BA-A6DB-01	C2
TCGA-CQ-A4CD-01	C2
TCGA-BA-4077-01	C2
TCGA-BA-4075-01	C2
TCGA-CV-7255-01	C2
TCGA-BA-A4IF-01	C2
TCGA-CV-A6JZ-01	C2
TCGA-BA-A6DJ-01	C2
TCGA-CN-6019-01	C2
TCGA-CN-4728-01	C2
TCGA-CV-5444-01	C2
TCGA-CQ-6222-01	C2
TCGA-F7-A50G-01	C2
TCGA-CQ-5332-01	C2
TCGA-C9-A47Z-01	C2
TCGA-CV-7434-01	C2
TCGA-QK-A8Z9-01	C2
TCGA-KU-A66T-01	C2
TCGA-CQ-6221-01	C2
TCGA-BA-6871-01	C2
TCGA-CR-6472-01	C2
TCGA-CN-6016-01	C2
TCGA-CQ-5326-01	C2

TCGA-IQ-A61J-01	C2
TCGA-CV-A45V-01	C2
TCGA-CV-7102-01	C2
TCGA-BB-4228-01	C2
TCGA-QK-A6V9-01	C2
TCGA-DQ-7592-01	C2
TCGA-CN-5366-01	C2
TCGA-QK-A6IG-01	C2
TCGA-UF-A7JO-01	C2
TCGA-CV-5435-01	C2
TCGA-CQ-6218-01	C2
TCGA-CV-7414-01	C2
TCGA-P3-A5QE-01	C2
TCGA-QK-A6IH-01	C2
TCGA-BA-4078-01	C2
TCGA-CV-7432-01	C2
TCGA-CQ-A4C6-01	C2
TCGA-CV-5979-01	C2
TCGA-CN-6997-01	C2
TCGA-DQ-7588-01	C2
TCGA-CN-4734-01	C2
TCGA-CV-7183-01	C2
TCGA-BA-5556-01	C2
TCGA-CV-7177-01	C2
TCGA-D6-A6EP-01	C2
TCGA-D6-6515-01	C2
TCGA-CV-7178-01	C2
TCGA-CV-6951-01	C2
TCGA-CQ-7069-01	C2
TCGA-CN-5364-01	C2
TCGA-CV-A6JT-01	C2
TCGA-HD-A6I0-01	C2
TCGA-CN-6998-01	C2
TCGA-CR-6491-01	C2
TCGA-P3-A6T3-01	C2
TCGA-CR-7365-01	C2
TCGA-CV-7097-01	C2
TCGA-UF-A7J9-01	C2
TCGA-CN-5367-01	C2
TCGA-CV-6960-01	C2
TCGA-BA-A6DE-01	C2
TCGA-CQ-7063-01	C2
TCGA-CR-7386-01	C2
TCGA-T3-A92N-01	C2
TCGA-CV-6934-01	C2
TCGA-CQ-7072-01	C2
TCGA-CN-6994-01	C2
TCGA-P3-A6T2-01	C2
TCGA-BA-4076-01	C2
TCGA-CN-5373-01	C2
TCGA-CR-6471-01	C2
TCGA-UF-A7JS-01	C2
TCGA-CV-A464-01	C2
TCGA-CQ-5330-01	C2
TCGA-CV-7253-01	C2
TCGA-CV-5442-01	C2
TCGA-CQ-A4CB-01	C2
TCGA-CV-7407-01	C2

TCGA-CR-7401-01	C2
TCGA-UF-A7JK-01	C2
TCGA-CQ-6229-01	C2
TCGA-BB-A6UM-01	C2
TCGA-CV-6940-01	C2
TCGA-CV-6956-01	C2
TCGA-CN-A641-01	C2
TCGA-BB-4227-01	C2
TCGA-CV-A45O-01	C2
TCGA-QK-A652-01	C2
TCGA-CV-A45Z-01	C2
TCGA-CV-7421-01	C2
TCGA-CV-A6JN-01	C2
TCGA-CV-5977-01	C2
TCGA-CN-6995-01	C2
TCGA-CV-6961-01	C2
TCGA-CV-7430-01	C2
TCGA-CV-6954-01	C2
TCGA-RS-A6TO-01	C2
TCGA-CN-A63V-01	C2
TCGA-CN-6011-01	C2
TCGA-CV-7245-01	C2
TCGA-CR-6488-01	C2
TCGA-CV-7104-01	C2
TCGA-CV-7090-01	C2
TCGA-CV-5971-01	C2
TCGA-CV-7089-01	C2
TCGA-D6-A6EN-01	C2
TCGA-P3-A6T6-01	C2
TCGA-UF-A7JA-01	C2
TCGA-CR-7383-01	C2
TCGA-D6-8569-01	C2
TCGA-MT-A67F-01	C2
TCGA-CV-7429-01	C2
TCGA-CV-7236-01	C2
TCGA-UF-A7JV-01	C2
TCGA-P3-A6T8-01	C2
TCGA-CV-A6K0-01	C2
TCGA-CV-6955-01	C2
TCGA-IQ-A61G-01	C2
TCGA-IQ-7630-01	C2
TCGA-IQ-A61O-01	C2
TCGA-BA-A4IH-01	C2
TCGA-T2-A6X2-01	C2
TCGA-BB-4225-01	C2
TCGA-F7-A61W-01	C2
TCGA-CV-7428-01	C2
TCGA-MT-A51W-01	C2
TCGA-CR-7376-01	C2
TCGA-BA-6872-01	C2
TCGA-CV-5434-01	C2
TCGA-CN-6024-01	C2
TCGA-CV-5966-01	C2
TCGA-WA-A7GZ-01	C2
TCGA-CQ-6219-01	C2
TCGA-UF-A7JC-01	C2
TCGA-CV-7437-01	C2
TCGA-CV-A461-01	C2

TCGA-CQ-5334-01	C2
TCGA-CV-7235-01	C2
TCGA-UF-A71A-01	C2
TCGA-QK-A6VB-01	C2
TCGA-CV-5976-01	C2
TCGA-BA-A6D8-01	C2
TCGA-CN-A642-01	C2
TCGA-HD-A634-01	C2
TCGA-CN-A49A-01	C2
TCGA-P3-A6T5-01	C2
TCGA-TN-A7HL-01	C2
TCGA-CN-A6V7-01	C2
TCGA-CR-7397-01	C2
TCGA-CV-7406-01	C2
TCGA-CQ-A4C7-01	C2
TCGA-F7-A50J-01	C2
TCGA-CN-4730-01	C2
TCGA-CV-A6K2-01	C2
TCGA-CR-5247-01	C2
TCGA-CN-4736-01	C2
TCGA-CV-7433-01	C2
TCGA-P3-A5QA-01	C2
TCGA-D6-6827-01	C2
TCGA-P3-A5QF-01	C2
TCGA-CV-6938-01	C2
TCGA-IQ-7631-01	C2
TCGA-MT-A67A-01	C2
TCGA-HD-8634-01	C2
TCGA-CR-6493-01	C2
TCGA-CV-7568-01	C2
TCGA-HD-A633-01	C2
TCGA-CV-7099-01	C2
TCGA-BA-A6DA-01	C2
TCGA-CV-7091-01	C2
TCGA-CN-A49B-01	C2
TCGA-UF-A7JH-01	C2
TCGA-CQ-6224-01	C2
TCGA-CV-A6JO-01	C2
TCGA-CV-5439-01	C2
TCGA-BA-5555-01	C2
TCGA-CN-A63W-01	C2
TCGA-CN-6989-01	C2
TCGA-CV-7247-01	C2
TCGA-CV-7422-01	C2
TCGA-CR-7372-01	C2
TCGA-CV-7103-01	C2
TCGA-BA-4074-01	C2
TCGA-DQ-5625-01	C2
TCGA-CR-7398-01	C2
TCGA-CV-7416-01	C2
TCGA-BA-6868-01	C2
TCGA-CN-5359-01	C2
TCGA-KU-A6H8-01	C2
TCGA-CQ-7068-01	C2
TCGA-CN-5365-01	C2
TCGA-CN-6022-01	C2
TCGA-CV-A6JE-01	C2
TCGA-CV-6933-01	C2

TCGA-CV-6950-01	C2
TCGA-P3-A5Q6-01	C2
TCGA-CV-7425-01	C2
TCGA-CQ-6220-01	C2
TCGA-D6-A6EM-01	C2
TCGA-HD-8224-01	C2
TCGA-CR-7382-01	C2
TCGA-CV-A6JD-01	C2
TCGA-T2-A6WX-01	C2
TCGA-CV-7446-01	C2
TCGA-CV-A6K1-01	C2
TCGA-CN-4731-01	C2
TCGA-CV-A6JU-01	C2
TCGA-CQ-A4CG-01	C2
TCGA-CN-A49C-01	C2
TCGA-BA-5557-01	C2
TCGA-HD-7832-01	C2
TCGA-F7-A624-01	C2
TCGA-H7-8501-01	C2
TCGA-CV-7427-01	C2
TCGA-CN-5370-01	C2
TCGA-CN-4737-01	C2
TCGA-F7-8298-01	C2
TCGA-DQ-5630-01	C2
TCGA-RS-A6TP-01	C2
TCGA-CQ-5331-01	C2
TCGA-CV-A45R-01	C2
TCGA-H7-8502-01	C2
TCGA-CV-5440-01	C2
TCGA-CN-6988-01	C2
TCGA-H7-7774-01	C2
TCGA-D6-6516-01	C2
TCGA-HL-7533-01	C2
TCGA-QK-A64Z-01	C2
TCGA-BA-7269-01	C2
TCGA-CQ-5333-01	C2
